# Clinical Attitudes Toward Tooth Preservation Versus Implant Therapy: Development and Preliminary Validation of a Questionnaire Among Early-Career Romanian Dentists

**DOI:** 10.3390/jcm15093299

**Published:** 2026-04-26

**Authors:** Vlad Constantin, Dragos Ioan Virvescu, Ionut Luchian, Florinel Cosmin Bida, Andrei Georgescu, Oana Maria Butnaru, Teona Ana-Maria Tudorici, Costin Iulian Lupu, Cristian Cojocaru, Dana Gabriela Budala

**Affiliations:** Grigore T. Popa University of Medicine and Pharmacy, 700115 Iasi, Romania

**Keywords:** periodontally compromised teeth, dental implants, questionnaire validation, Cronbach’s alpha, exploratory factor analysis

## Abstract

**Background/Objectives**: The clinical decision between preserving periodontally compromised teeth and replacing them with dental implants represents a complex clinical dilemma influenced by biological, prosthetic, economic, and professional factors. The aim of this pilot study was to develop and preliminarily validate a questionnaire designed to assess dentists’ attitudes and therapeutic preferences regarding the use of periodontally compromised teeth as prosthetic abutments versus extraction and implant-supported rehabilitation. **Methods**: An observational cross-sectional study was conducted, among Romanian dentists, using a structured self-administered questionnaire consisting of 43 items organized into seven sections addressing clinical attitudes, decision-making factors, professional competence, prosthetic treatment preferences, and implant-related clinical practices. A total of 111 Romanian dentists completed the questionnaire. Responses were recorded using a five-point Likert scale. Statistical analysis was performed using IBM SPSS Statistics software. Internal consistency was evaluated using Cronbach’s alpha coefficient and intraclass correlation coefficient (ICC). Construct validity was assessed using exploratory factor analysis based on Principal Component Analysis with Varimax rotation. **Results**: The questionnaire demonstrated good internal consistency across most sections, with Cronbach’s alpha values ranging between 0.795 and 0.859 after scale optimization. Item–total correlations indicated adequate contribution of individual items to overall scale reliability. Intraclass correlation coefficients confirmed moderate reliability for individual items and good reliability for average section scores. Exploratory factor analysis showed satisfactory sampling adequacy (KMO = 0.709) and statistically significant Bartlett’s test of sphericity (*p* < 0.001), supporting the suitability of the data for factor analysis. The sample population was predominantly composed of early-career dentists with limited clinical experience, which should be considered when interpreting the findings. **Conclusions**: The developed questionnaire demonstrated satisfactory psychometric properties, including good internal consistency and acceptable construct validity, supporting its use as a research instrument for assessing Romanian dentists’ self-reported attitudes, therapeutic preferences, and perception-based decision patterns regarding the preservation of periodontally compromised teeth and implant-supported prosthetic rehabilitation.

## 1. Introduction

In contemporary dental practice, clinicians are frequently confronted with the complex decision of whether a compromised tooth should be preserved through restorative or periodontal treatment or replaced with a dental implant [1]. Advances in implant dentistry have significantly expanded the therapeutic options available to clinicians, offering predictable outcomes and high long-term survival rates. Consequently, dental implants are increasingly considered a reliable solution for the replacement of missing teeth [1,2]. However, the decision to extract a tooth and replace it with an implant is not always straightforward and must be carefully balanced against the potential benefits of maintaining the natural dentition.

Tooth preservation remains a fundamental principle of dentistry. Numerous studies emphasize that maintaining natural teeth, when feasible, is often associated with favorable biological, functional, and economic outcomes [3,4]. Improvements in periodontal therapy, endodontic treatment, and restorative materials have enhanced the prognosis of teeth that were previously considered questionable or hopeless, allowing clinicians to extend the longevity of natural dentition in many clinical situations [3,4,5]. Consequently, the choice between preserving a compromised tooth and replacing it with an implant requires careful evaluation of several factors, including periodontal status, endodontic condition, structural integrity of the tooth, patient systemic health, and long-term prognosis [6,7].

In addition to clinical parameters, dentists’ perceptions, experience, and professional attitudes may influence self-reported therapeutic preferences and perception-based decision tendencies. The increasing popularity of implant therapy, differences in professional training, evolving treatment philosophies, and economic considerations may contribute to variations in treatment recommendations among clinicians [8,9]. As a result, the same clinical situation may lead to different therapeutic decisions depending on the clinician’s background, experience, and interpretation of the available evidence.

Understanding dentists’ perspectives regarding tooth preservation versus implant replacement is therefore essential for evaluating current trends in clinical practice and identifying potential differences in decision-making patterns.

The question of whether a compromised tooth should be preserved or extracted and replaced with an implant has already been discussed in the literature from both biological and decision-oriented perspectives. Earlier reviews emphasized that treatment planning in such cases must integrate prognosis, restorability, periodontal status, patient-related factors, and long-term maintenance considerations rather than assuming the superiority of one therapeutic option over the other [10,11,12].

Questionnaire- and scenario-based studies have further shown that dentists’ recommendations are not uniform and may vary according to specialty training, professional experience, and individual interpretation of clinical findings. In particular, Azarpazhooh et al. reported variability in dentists’ preferences for the management of teeth with apical periodontitis, while Bigras et al. demonstrated differences in clinical attitudes between specialists and general dentists [13,14]. At the same time, Lee et al. showed that specialty and work experience influenced extraction-related decisions and Alamri found substantial variation in dentists’ choices when presented with clinical scenarios involving tooth preservation versus implant replacement [15,16]. Taking together, these studies indicate that the topic is clinically relevant and already explored, but the available evidence remains methodologically heterogeneous and lacks a standardized validated instrument specifically designed to assess these attitudes in the present professional context. Moreover, standardized tools designed to evaluate dentists’ perceptions and decision-making processes in this context remain limited [17].

For this reason, the present pilot study aims to develop and preliminarily validate a questionnaire designed to assess dentists’ self-reported attitudes and perception-based therapeutic preferences regarding the preservation of compromised teeth versus their replacement with dental implants.

By exploring clinicians’ perceptions and treatment preferences, this study seeks to provide initial insights into how dentists approach this important therapeutic dilemma and to establish a methodological basis for future research involving larger and more diverse professional populations.

## 2. Materials and Methods

The present study was conducted using a structured methodological framework designed to ensure the reliability and reproducibility of the research process. The methodological approach was established prior to data collection and included the definition of the study design, the development of the research instrument, the selection of participants, and the procedures used for data analysis. Particular attention was given to the clarity and consistency of the research protocol in order to obtain valid and interpretable results.

The methodological structure of the study followed commonly accepted principles for questionnaire-based research in healthcare sciences. This approach aimed to ensure that the data obtained accurately reflected the perspectives and decision-making processes of the participants. Furthermore, methodological procedures were organized to allow transparent reporting and facilitate the interpretation of the study outcomes within the broader scientific context.

✓ Study design

An observational cross-sectional study was conducted using a structured, self-administered questionnaire aimed at assessing Romanian dentists’ perceptions, attitudes, and self-reported perceptions and therapeutic preferences regarding the use of periodontally compromised teeth as prosthetic abutments versus tooth extraction and subsequent implant-supported prosthetic rehabilitation.

In this initial stage, the questionnaire was developed with input from clinicians experienced in periodontology to ensure clinical relevance; however, a formal content validation process was not performed, which represents a limitation and should be addressed in future studies. Also, test–retest reliability was not assessed in the present study; therefore, the temporal stability of the instrument remains to be established in future research.

The questionnaire was originally developed and administered in Romanian. The English version presented in this manuscript is a translated version provided for reporting purposes only.

✓ Participants and Data Collection

The questionnaire was distributed to Romanian practicing dentists from various dental specialties and participation in the study was voluntary and anonymous, and no personally identifiable information was collected. The questionnaire was administered anonymously using Survio^®^ (Brno, Czech Republic) platform with its’ available technical submission controls. Therefore, duplicate or ineligible responses cannot be entirely excluded and should be considered when interpreting the findings.

Prior to completing the questionnaire, participants were informed about the purpose of the study, the confidentiality of the collected data, and the fact that the information would be used exclusively for scientific research purposes. Completion of the questionnaire was considered equivalent to providing informed consent for participation in the study.

No data regarding the total number of invitations distributed were available; therefore, a response rate could not be calculated. No financial or material incentives were provided, and no structured reminder system was implemented.

✓ Research Instrument

Data were collected using a questionnaire specifically developed for the purposes of this study. The instrument was structured into seven sections and included a total of 43 items. The first section collected general and professional information about the participants, while the subsequent sections assessed dentists’ attitudes, perceptions, and clinical practices related to the study topic.

With the exception of the descriptive section, all items were formulated as statements and evaluated using a five-point Likert scale, where 1 = strongly disagree, 2 = disagree, 3 = neutral, 4 = agree, and 5 = strongly agree. Some items were intentionally framed to reflect clinically realistic decision-making contexts, which may favor professionally desirable responses and introduce a degree of response bias.

✓ Questionnaire Structure

Section 1—General and Professional Characteristics (5 items)

This section collected descriptive information regarding dental specialization, years of professional experience, practice setting (urban/rural), type of professional activity (private practice, multidisciplinary clinic, public sector, or academic environment), and participation in continuing medical education programs. These variables were used exclusively for descriptive and comparative analyses and were not included in the attitudinal scoring of the questionnaire.

Section 2—Attitudes Regarding the Use of Periodontally Compromised Teeth as Prosthetic Abutments (4 items)

This section assessed dentists’ conservative clinical attitudes toward preserving periodontally compromised teeth and incorporating them into prosthetic restorations. It also explored the clinical conditions considered necessary for adopting this therapeutic approach, such as adequate periodontal treatment, periodontal stabilization, and patient compliance with supportive therapy and oral hygiene recommendations.

Section 3—Attitudes Toward Tooth Extraction and Implant Therapy (5 items)

This section examined dentists’ preferences regarding tooth extraction followed by implant placement. The items explored clinicians’ perceptions of the long-term predictability of implant-supported restorations, the perceived efficiency of implant therapy, and the comparative evaluation of risks associated with maintaining compromised teeth versus replacing them with dental implants.

Section 4—Determinants in Prosthetic Treatment Planning (6 items)

This section explored the factors influencing therapeutic decision-making in prosthetic treatment planning. Specifically, it assessed the impact of the individual tooth prognosis, patient age, financial considerations, patient expectations, and interdisciplinary collaboration (e.g., with periodontists or other dental specialists) on clinicians’ treatment choices.

Section 5—Professional Competence and Training (7 items)

This section evaluated dentists’ perceived clinical competence in assessing the viability of periodontally compromised teeth and their confidence in prosthetic treatment planning. The items also addressed the adequacy of previous professional training, the level of familiarity with and application of current clinical guidelines (including the EFP S3 guideline and the 2018 periodontal classification), as well as the respondents’ interest in further professional education and training in this field. Specialty was recorded based on self-reported data, without additional information regarding the timing of certification or level of training. As a result, some respondents classified as specialists may have been in early stages of their professional development. This may represent a confounding factor, particularly in relation to the assessment of professional competence. Future studies should include more detailed variables regarding professional training and certification status in order to improve the precision of subgroup characterization.

Section 6—Preferred Prosthetic Restorative Approaches in Cases with Reduced Periodontal Support (6 items)

This section examined the prosthetic treatment strategies commonly used in clinical practice when managing cases with reduced periodontal support. The items explored clinicians’ preferences regarding fixed restorations, combined fixed–removable prosthetic solutions, provisional restorations, tooth–implant-supported rehabilitations, as well as the tendency to avoid using teeth with severe periodontal support loss as prosthetic abutments.

Section 7—Implant-Supported Prosthetic Restorations: Attitudes and Clinical Practices (10 items)

This section assessed clinicians’ attitudes and clinical practices regarding implant-supported prosthetic restorations. The items explored dentists’ preferences for implant-based rehabilitation, perceptions of long-term predictability, indications for fixed and removable implant-supported restorations, acceptance of combined tooth–implant abutment designs, and the influence of previous clinical experiences (e.g., peri-implantitis) on therapeutic decision-making.

✓ Questionnaire Distribution

Using the Survio^®^ platform (Brno, Czech Republic), the questionnaire was made available online for two months after distribution. Various online communication channels often utilized by the target group were employed to promote the survey link.

Everyone was free to participate in an anonymous manner, and they could fill out the survey whenever it was convenient for them. Before participants could access the survey questions, they were briefed about the study’s goals, how their replies would be handled in a confidential manner, and how the data would only be used for academic research. No personal identifiers were collected during the survey.

Ethical principles governing biomedical research were respected throughout the study, in accordance with the Declaration of Helsinki. Ethical approval was granted by the Ethics Committee of the Grigore T. Popa University of Medicine and Pharmacy in Iasi, Romania, protocol code 692/22.12.2025. By submitting the complete questionnaire, participants implicitly expressed their informed consent to take part in the research. “Ethical approval was obtained on 22 December 2025, and data collection was conducted over a two-month period thereafter. Given the pilot nature of the study and characteristics of Survio^®^ (Brno, Czech Republic) platform, this timeframe was considered appropriate.

The online administration of the survey ensured consistent distribution of the questionnaire and facilitated efficient data acquisition. Only responses corresponding to fully completed questionnaires were retained and subsequently included in the statistical evaluation.

✓ Scoring and Interpretation

The questionnaire consisted of 43 items organized into seven sections. Section 1 included descriptive variables related to participants’ characteristics and was not included in the psychometric analyses. Only Sections 2–7 were subjected to internal consistency and construct validity evaluation. In addition, certain items were excluded during the scale optimization process based on psychometric criteria, which resulted in a reduced number of items included in the final analysis. For Sections 2–7, responses were numerically coded on a scale from 1 to 5. Mean scores for each section were calculated by dividing the total score of the items by the number of items included in that section. Higher scores indicated a greater level of agreement with the construction being evaluated.

The interpretation of the mean scores was defined as follows: the midpoint value (3.00) was considered indicative of a neutral response. Values below this point reflect a tendency toward disagreement, while values above indicate a tendency toward agreement. Scores close to the midpoint should be interpreted cautiously, as they may reflect weak tendencies or variability rather than clearly defined attitudes.

Based on the obtained scores, respondents could be categorized into indicative profiles of clinical decision-making, such as conservative-oriented, implant-oriented, or interdisciplinary-oriented approaches.

✓ Statistical Analysis and Instrument Validation

Statistical analysis was performed using IBM SPSS Statistics, version 29.0.0 (IBM Corp., Armonk, NY, USA). The outcomes derived from the applied statistical analyses are reported with reference to sample characteristics and the psychometric properties of the instrument.

✓ Instrument Reliability

The internal consistency of the questionnaire and its subscales (Sections 2–7) was assessed using Cronbach’s alpha coefficient, with values ≥ 0.70 considered acceptable for exploratory research. Item–total correlation analysis was also performed to evaluate the contribution of each item to the overall internal consistency and to identify potential opportunities for optimizing specific sections of the questionnaire.

In addition, the reliability of the composite scores was further examined by calculating the intraclass correlation coefficient (ICC). A two-way mixed-effects model with a consistency definition was applied in order to assess the stability and homogeneity of the measurements at the section level. The intraclass correlation coefficient (ICC) values obtained for Single Measures indicate a moderate level of agreement at the individual item level, suggesting variability in responses across items. In contrast, the ICC values for Average Measures reflect higher reliability at the composite score level.

✓ Construct Validity

Construct validity was assessed using exploratory factor analysis (EFA) based on the Principal Component Analysis (PCA) method. Principal Component Analysis (PCA) was used as the extraction method, resulting in initial communality values of 1.000 for all items. This approach was selected for exploratory purposes, focusing on data reduction and identification of underlying structure. The suitability of the dataset for factor analysis was first evaluated using the Kaiser–Meyer–Olkin (KMO) measure of sampling adequacy and Bartlett’s test of sphericity, with a significance level of *p* < 0.05 indicating sufficient correlations among items to justify the application of factor analysis. Reliability was assessed using the Intraclass Correlation Coefficient (ICC), based on a two-way mixed-effects model with consistency definition.

The number of retained factors was determined using the eigenvalue > 1 criterion, together with the examination of the total variance explained and the visual inspection of the Scree Plot. To facilitate interpretation of the factor structure, Varimax rotation with Kaiser normalization was applied. The resulting factorial solution was evaluated by analyzing factor loadings, communalities, and the graphical representation of variables within the rotated component space. The number of retained factors was determined based on the Kaiser criterion (eigenvalues > 1) and supported by visual inspection of the scree plot. Given the exploratory nature of the study and the relatively limited sample size, the adequacy of the factor analysis should be interpreted with caution. Although the KMO value supported the application of factor analysis, sample adequacy cannot be assessed solely based on this index.

In particular, the subject-to-item ratio in the present study remains below commonly recommended thresholds, which may affect the stability of the extracted factor structure. Moreover, the relatively high number of extracted components in relation to the number of retained items suggests that some factors may be defined by a limited number of items, which may reduce their psychometric stability and interpretability. Therefore, the results of the exploratory factor analysis should be considered preliminary and require confirmation in larger samples, ideally through confirmatory factor analysis (CFA).

## 3. Results

*Section 1—Distribution of Participants According to Specialty*

A total of 111 dentists completed the questionnaire and were included in the analysis. The descriptive analysis of the variable specialty indicates a heterogeneous distribution of respondents, with a predominance of dentists without a declared specialty. More than half of the participants (54.1%) reported being general dental practitioners, representing the largest segment of the sample. Periodontology specialists constituted the second most represented group (23.4%), followed by dentists specializing in prosthodontics (9.9%). A smaller proportion of respondents consisted of specialists in general dentistry (4.5%), while 8.1% of participants indicated another specialty not included among the predefined categories in the questionnaire. Although the study included dentists from various specialties, the distribution across subgroups was uneven, with relatively small sample sizes in certain categories. Therefore, the study does not have sufficient statistical power to support reliable comparisons between specialties, and such analyses should be interpreted with caution.

The analysis of respondents’ distribution by professional experience indicates a predominance of dentists with relatively limited clinical experience. Overall, the sample was predominantly composed of early-career dentists, indicating a limited level of clinical experience across the majority of respondents. Nearly three quarters of the participants (73.9%) reported having less than five years of professional practice, representing the largest segment of the study sample. A proportion of 21.6% of respondents reported a professional experience between five and ten years, suggesting a notable representation of dentists in the intermediate stage of their professional careers.

In contrast, participants with longer professional experience were considerably underrepresented. Only 0.9% of the respondents reported having between 11 and 20 years of practice, while 3.6% indicated more than 20 years of professional activity.

The analysis of the variable related to the professional practice setting indicates a distribution clearly oriented toward the urban environment. The vast majority of respondents (93.7%) reported working in dental facilities located in urban areas, whereas only a small proportion (6.3%) indicated practicing in rural settings. This distribution suggests that the study sample is predominantly composed of dentists practicing in urban healthcare environments.

The analysis of the primary workplace organization indicates a predominance of professional activity within private multidisciplinary structures. Nearly half of the respondents (48.6%) reported working in private multidisciplinary clinics, making this the most frequently reported type of professional setting. Individual dental practices represented the second most common category, being indicated by 32.4% of participants. At the same time, the academic environment accounted for 18.9% of respondents, suggesting a meaningful participation of professionals involved in both teaching and clinical activities.

The analysis of the annual participation frequency in professional courses and scientific congresses indicates a relatively high level of engagement in continuing professional education. The majority of respondents (51.4%) reported attending one or two scientific events per year, representing the largest segment of the sample. A considerable proportion of participants (31.5%) indicated a higher level of involvement, participating in three or more courses or congresses annually. In contrast, 17.1% of respondents reported not attending any such activities within a year, suggesting the presence of a subgroup with limited access to or interest in continuing professional development.

*Section 2* *—Attitudes Regarding the Use of Periodontally Compromised Teeth as Prosthetic Abutments*

The internal consistency of the first section of the questionnaire was evaluated using Cronbach’s alpha coefficient. The obtained value (α = 0.809) indicates a good level of internal reliability, suggesting adequate homogeneity of the included items and satisfactory coherence of the scale in assessing attitudes toward the use of periodontally compromised teeth as prosthetic abutments (Table 1).

The section consists of four items that recorded relatively high mean values, ranging from 4.44 to 4.70, reflecting a strong level of agreement among respondents with the statements presented. The dispersion of responses was relatively limited, with standard deviations varying between 0.46 and 0.72, indicating moderate variability and good consistency in the expressed opinion.

The items address complementary aspects of a conservative therapeutic approach, including the viability of maintaining periodontally compromised teeth within prosthetic rehabilitation, the preference for preserving natural teeth rather than opting for extraction and implant placement, as well as the acceptability of incorporating teeth with controlled periodontal mobility into prosthetic restorations under conditions of adequate periodontal treatment and patient compliance. The conceptual coherence of these items supports the reliability coefficient obtained for this section.

The descriptive statistics for the items included in Section 2 are presented in Table 2. The results indicate generally high levels of agreement with the statements assessing attitudes toward the use of periodontally compromised teeth as prosthetic abutments.

Overall, the results indicate that this section of the questionnaire demonstrates adequate internal consistency and can be considered reliable for assessing dentists’ attitudes toward the use of periodontally compromised teeth as prosthetic abutments in clinical prosthetic practice.

The item–total statistics further reveal an appropriate level of correlation between each individual item and the overall scale score. Corrected item–total correlation coefficients ranged between 0.55 and 0.71, exceeding the commonly accepted minimum threshold in psychometric evaluations. This finding suggests that each item contributes meaningfully to the overall construction measured within this section.

The item addressing the acceptability of incorporating teeth with Grade I–II periodontal mobility into prosthetic restorations showed the highest corrected item–total correlation (r = 0.711), indicating a strong association with the total scale score. The remaining items also demonstrated consistent correlations, reflecting good conceptual alignment among the statements included in this section.

Furthermore, Cronbach’s alpha values calculated in the case of item deletion were lower than the overall alpha coefficient obtained for the full scale (α = 0.809), ranging between 0.720 and 0.795. This result indicates that the removal of any item would not improve the internal consistency of the scale, thereby supporting the retention of all items in the final structure of this questionnaire section.

The item–total statistics for Section 2 are presented in Table 3. These results provide additional evidence regarding the internal consistency of the scale by examining the contribution of each individual item to the overall reliability of the section.

Analysis of variance (ANOVA) was performed to examine differences among the items included in the first attitudinal section of the questionnaire based on the scores provided by respondents. The results revealed statistically significant differences between the items, as indicated by the F-test value (F = 3.660; *p* = 0.014).

The variance attributed to differences between items (mean square = 0.684) was higher than the residual variance (mean square = 0.187), suggesting that the items were not evaluated identically by respondents but rather captured distinct aspects of the attitudes under investigation. This finding is consistent with a well-structured scale in which individual items, although correlated, contribute differentially to the assessment of the underlying construct.

The overall mean score (Grand Mean = 4.54) further confirms the high level of agreement with the statements included in this section, supporting the results obtained from both the descriptive statistics and the internal consistency analysis.

Overall, the ANOVA results support the section’s ability to discriminate between different dimensions of attitudes regarding the use of periodontally compromised teeth as prosthetic abutments, without compromising the overall coherence of the scale. These findings further strengthen the evidence for the reliability and construct validity of the instrument.

The results of the analysis of variance (ANOVA) for the items included in Section 2 are presented in Table 4. This analysis was conducted to examine whether significant differences exist between the mean scores of the items assessing attitudes toward the use of periodontally compromised teeth as prosthetic abutments. The analysis revealed statistically significant differences between items (F = 3.660, *p* = 0.014).

The reliability of the assessment performed through the items included in the first attitudinal section of the questionnaire was further examined by calculating the Intraclass Correlation Coefficient (ICC). A two-way mixed-effects model was applied, in which the effects associated with respondents were considered random, while the effects associated with the items were treated as fixed. A consistency definition was used, which excludes variance between measurements from the denominator of the total variance.

For individual measurements (Single Measures), the ICC value obtained was 0.515, with a 95% confidence interval ranging from 0.380 to 0.648, indicating a moderate level of agreement at the individual item level and suggesting variability in responses across items.

The associated F-test was statistically significant (F = 5.240; *p* < 0.001), confirming that the ICC values are significantly different from zero. This finding indicates the presence of a genuine level of consistency among the items of the scale beyond random variation.

Overall, the intraclass correlation analysis confirms the stability and internal coherence of Section 1 of the questionnaire, supporting its reliability in assessing attitudes regarding the use of periodontally compromised teeth as prosthetic abutments. The intraclass correlation coefficient (ICC) was calculated to further assess the reliability of the scale used in Section 2 of the questionnaire. The results of the ICC analysis are presented in Table 5.

*Section 3* *—Attitudes Regarding Tooth Extraction and Dental Implants*

The internal consistency of Section 3 of the questionnaire was evaluated using Cronbach’s alpha coefficient. The obtained value (α = 0.853) indicates a good to very good level of internal reliability, suggesting a high degree of coherence among the items included in this dimension.

This section consists of five items designed to assess dentists’ attitudes toward tooth extraction and the use of dental implants as a therapeutic option. The obtained alpha coefficient indicates that the items are well correlated with each other and contribute consistently to the measurement of the same underlying construct (Table 6).

The descriptive statistical analysis of the items included in Section 3 revealed mean values within a moderate range, varying between 2.28 and 3.46, suggesting greater variability in respondents’ opinions regarding attitudes toward tooth extraction and the use of dental implants.

The highest mean values were recorded for the statements supporting the preference for dental implants in cases of severe periodontal involvement (M = 3.46) and the perceived long-term stability of implants compared with the maintenance of periodontally compromised teeth (M = 3.30). These findings indicate a moderate level of acceptance of implant-based solutions in complex clinical situations.

In contrast, the item referring to the preference for tooth extraction and implant placement even in situations where the tooth could theoretically be preserved recorded the lowest mean value (M = 2.28), reflecting respondents’ reluctance toward more radical approaches and suggesting a relatively cautious attitude in therapeutic decision-making.

Standard deviation values ranging between 0.96 and 1.22 indicate a moderate dispersion of responses, suggesting the presence of differing perspectives among respondents. This variability is consistent with the nature of the topic under investigation, which involves clinical decisions influenced by multiple factors, including disease severity, professional experience, and the specific clinical context.

Descriptive statistics of participants’ perceptions on implant placement versus periodontal treatment are summarized in Table 7.

The item–total statistics indicate an appropriate correlation between each item and the overall score of Section 3. Corrected item–total correlation values ranged from 0.528 to 0.778, exceeding the commonly accepted minimum threshold for reliability assessments. This finding suggests that all items contribute meaningfully to the measurement of the construction evaluated in this section.

The highest item–total correlations were observed for the statements referring to the long-term stability of dental implants compared with the maintenance of periodontally compromised teeth (r = 0.740) and to the perceived risk of peri-implantitis relative to the risk of subsequent loss of teeth affected by periodontal disease (r = 0.778). These results indicate a strong alignment of these items with the overall scale score.

Cronbach’s alpha values calculated in the case of item deletion ranged between 0.794 and 0.858. The removal of any individual item did not result in a meaningful improvement in the internal consistency compared with the alpha coefficient obtained for the entire section (α = 0.853). Although excluding the first item would produce a slightly higher alpha value (α = 0.858), the difference is minimal and does not justify its removal from either a psychometric or conceptual perspective.

Overall, these findings support the retention of all items in the structure of Section 3, confirming the internal coherence of the scale and the relevance of each statement in assessing dentists’ attitudes toward tooth extraction and the use of dental implants. A detailed overview of the item–total statistics is provided in Table 8.

Analysis of variance (ANOVA) was conducted to evaluate differences among the items included in Section 3 of the questionnaire. The results revealed statistically significant differences between the items, with a relatively high F-test value (F = 20.743; *p* < 0.001).

The variance associated with differences between items (Mean Square = 11.139) was substantially higher than the residual variance (Mean Square = 0.537), suggesting that the statements included in this section are perceived differently by respondents and capture distinct perspectives regarding clinical decisions related to tooth extraction and implant therapy.

The overall mean score (Grand Mean = 3.00) indicates a neutral response pattern among participants toward the analyzed statements, which may reflect either a lack of clear preference or variability in respondents’ perceptions, as also suggested by the distribution of individual item means and the observed variability in the descriptive analysis.

The statistically significant differences identified between items do not indicate a lack of coherence of the scale; rather, they support its ability to discriminate between different facets of the attitudes under investigation, as illustrated in Table 9. This aspect contributes to strengthening the internal validity of Section 3.

The reliability of Section 3 of the questionnaire was further evaluated by calculating the Intraclass Correlation Coefficient (ICC). A two-way mixed-effects model was applied, in which the effects associated with respondents were considered random, while the effects associated with the items were treated as fixed. A consistency definition was used, which excludes variance between measurements from the calculation of the total variance.

For individual measurements (Single Measures), the ICC value was 0.537, with a 95% confidence interval ranging from 0.415 to 0.661, indicating a moderate level of agreement at the individual item level and suggesting variability in responses across items.

For the average measurements (Average Measures), the ICC reached a value of 0.853, with a 95% confidence interval between 0.780 and 0.907, indicating a moderate level of agreement at the individual item level and suggesting variability in responses across items. This value is consistent with the Cronbach’s alpha coefficient obtained for Section 3 and supports the use of the mean item score in subsequent analyses.

Overall, the results of the intraclass correlation coefficient analysis illustrated in Table 10 confirm the stability and internal coherence of Section 3 of the questionnaire, supporting its reliability in assessing attitudes toward tooth extraction and the use of dental implants.

The associated F-test was statistically significant (F = 6.802; *p* < 0.001), confirming that the ICC values are significantly different from zero and indicating the presence of a genuine level of consistency among the items included in this section.

*Section 4* *—Decision-Making Factors in Prosthetic Treatment Planning*

The internal consistency of Section 4 of the questionnaire was assessed using Cronbach’s alpha coefficient. The obtained value (α = 0.859) indicates a good level of internal reliability, suggesting a high degree of coherence among the items included in this dimension.

This section comprises six items designed to investigate the factors involved in prosthetic treatment decision-making, encompassing clinical, biological, and behavioral considerations. The value of the alpha coefficient indicates that the items are well correlated with one another and consistently contribute to the measurement of the same underlying construction, without suggesting excessive redundancy.

The obtained result supports the stability and internal homogeneity of this section, indicating that the instrument is appropriate for analyzing the factors influencing prosthetic treatment planning. Therefore, Section 4 can be considered reliable and suitable for use in subsequent statistical analyses (Table 11).

The descriptive statistical analysis of the items included in Section 4 revealed relatively high mean values, ranging from 3.80 to 4.26, indicating a generally high level of agreement among respondents regarding the proposed determinants of prosthetic treatment decision-making.

The highest mean value was recorded for the item referring to the influence of financial costs on the final treatment decision (M = 4.26), highlighting the recognition of economic factors as an important element in clinical practice. Additionally, the individual tooth prognosis—evaluated based on clinical parameters such as mobility, attachment level, and bone support—was considered a key determinant in therapeutic decision-making (M = 4.13).

Items addressing interdisciplinary collaboration, both with periodontists and within a broader clinical team, showed comparable mean values (M ≈ 4.13–4.19), suggesting a consistent appreciation of the importance of an interdisciplinary approach in prosthetic treatment planning. In contrast, the influence of patient age and the role of patient expectations demonstrated slightly lower mean values (M ≈ 3.80–3.87), indicating greater variability in respondents’ perspectives regarding these factors.

The standard deviations, ranging between 0.62 and 0.98, reflect a moderate dispersion of responses, without indicating extreme values or major inconsistencies among items. This distribution suggests that the statements were well calibrated and adequately captured the relevant factors involved in prosthetic decision-making. Descriptive statistics of the items included in Section 4 are presented in Table 12.

The item–total statistics analysis revealed adequate correlations between each item and the overall score of Section 4. The corrected item–total correlation coefficients ranged from 0.599 to 0.781, exceeding the minimum acceptable threshold for reliability assessment and indicating that all items meaningfully contribute to the measurement of the underlying construct.

The highest item–total correlations were observed for items related to collaboration with a periodontist (r = 0.781) and interdisciplinary collaboration (r = 0.717), suggesting a strong association between these aspects and the overall scale score. These findings further emphasize the critical role of interdisciplinary approaches in prosthetic decision-making.

Cronbach’s alpha values calculated upon deletion of individual items were lower than the overall alpha coefficient for the entire section (α = 0.859), ranging between 0.815 and 0.847. This result indicates that the removal of any item would not improve the internal consistency of the scale, thereby supporting the retention of all items in the final structure of Section 4. Overall, the item–total statistics confirm the internal coherence of this section and the relevance of each item in assessing factors influencing prosthetic treatment decision-making, thus strengthening the reliability argument of the questionnaire, as illustrated in Table 13.

Analysis of variance (ANOVA) was performed to assess differences among the items included in Section 4 of the questionnaire. The results revealed statistically significant differences between items, with an F value of 5.920 (*p* < 0.001).

Comparison of mean square values showed that the variance associated with differences between items (Mean Square = 1.842) exceeded the residual variance (Mean Square = 0.311), indicating that the statements within this section are perceived differently by respondents and capture distinct dimensions of prosthetic decision-making.

The overall mean score (Grand Mean = 4.06) reflects a generally high level of agreement with the proposed factors, confirming their relevance in clinical practice and aligning with the findings of the descriptive item analysis.

The ANOVA results for Section 4 items indicate statistically significant differences between items (F = 5.920, *p* < 0.001), as detailed in Table 14.

The reliability of Section 4 of the questionnaire was further evaluated using the Intraclass Correlation Coefficient (ICC), based on a two-way mixed-effects model, where respondent effects were considered random and item effects fixed. A consistency definition was applied, excluding between-measurement variance from the total variance estimation.

For single measurements (Single Measures), the ICC value was 0.504, with a 95% confidence interval ranging from 0.388 to 0.628, indicating a moderate level of agreement at the individual item level and suggesting variability in responses across items.

For average measurements (Average Measures), the ICC reached 0.859, with a 95% confidence interval between 0.792 and 0.910, indicating good reliability of the composite scale score. This finding is consistent with the Cronbach’s alpha coefficient obtained for this section and supports the use of the mean item score in subsequent analyses.

The associated F-test was statistically significant (F = 7.101; *p* < 0.001), confirming that the ICC values differ significantly from zero and indicating the presence of true consistency among the items. The intraclass correlation analysis demonstrated moderate reliability for single measures and good reliability for average measures, as presented in Table 15.

The internal consistency of the scale was initially assessed using Cronbach’s alpha, with the obtained value falling below the threshold generally considered acceptable for psychometric analyses. This result indicated insufficient homogeneity among the items and suggested the need to revise the initial structure of the scale.

Following an evaluation of item performance and their conceptual coherence, several items were removed and adjustments were made to the scale formulation in order to improve internal consistency. After these modifications, Cronbach’s alpha increased substantially, reaching a value of 0.819, which exceeds the acceptable threshold and indicates adequate coherence among the remaining items (Table 16).

The comparative analysis of descriptive statistics for the items included in the initial and adjusted versions of the scale highlights relevant changes both in item selection and in score distribution. Following the internal consistency optimization process, three items were removed, and four statements were retained in the final structure of the scale.

For the retained items, mean values generally increased after adjustment, suggesting improved alignment with the underlying construction. For instance, the item referring to perceived competence in assessing the viability of a periodontally compromised tooth showed an increase in mean score from 3.91 to 4.13, accompanied by a slight reduction in response dispersion. A similar pattern was observed for the item related to training in the preservation of teeth with compromised periodontium, where the mean value increased from 3.22 to 3.80.

The item addressing the perception of gaps in clinical practice guidelines also demonstrated a moderate increase in mean score after adjustment, while maintaining comparable variability in responses. Regarding interest in additional training, mean values remained high in both stages, with only minor variations in dispersion, indicating relative stability of this dimension.

The removal of items related to confidence in general clinical skills, the use of the S3 guideline of the European Federation of Periodontology, and the application of the 2018 EFP classification contributed to reducing the heterogeneity of the scale. Although conceptually relevant, these items exhibited greater variability and weaker alignment with the final construct structure. Item removal was based on a combination of statistical and conceptual criteria, including low item–total correlations, weak factor loadings, and limited contribution to the interpretability of the construct. Given the exploratory nature of the study, these decisions were applied iteratively and were not fully pre-specified.

Overall, the observed changes in mean values and standard deviations, together with the improvement in Cronbach’s alpha, indicate that the adjusted version of the scale is more homogeneous and psychometrically coherent, supporting its use in subsequent analyses. The descriptive statistical analysis of the questionnaire items, including mean values and standard deviations before and after adjustment, is presented in Table 17.

Item–total statistics analysis for the initial version of the scale revealed unequal contributions of the items to the overall internal consistency. The corrected item–total correlation values varied considerably, ranging from negative values to moderate correlations, suggesting an inconsistent alignment of some items with the construct measured by the scale.

In the initial version, the item referring to the existence of gaps in clinical guidelines regarding the decision between extraction and tooth preservation showed a negative item–total correlation (r = −0.173), indicating an inverse relationship with the total scale score. Additionally, the items addressing interest in additional training and the level of training in the preservation of periodontally compromised teeth showed low item–total correlations (r = 0.157 and r = 0.324, respectively), suggesting a limited contribution to the homogeneity of the scale in its initial form.

The Cronbach’s alpha values if items were deleted in the initial version ranged between 0.440 and 0.656, failing to reach the acceptable threshold for internal consistency. These results indicated the need to revise the scale structure and reconsider item selection.

Following the adjustment process, which involved removing items with low psychometric performance and retaining those that were conceptually and statistically better aligned with the construct, the Cronbach’s alpha values if items were deleted for the remaining items increased substantially, ranging between 0.708 and 0.798. This increase reflects a significant improvement in internal consistency and a better coherence among the items included in the final version of the scale.

The retained items showed improved item–total correlations and a positive contribution to the stability of the scale, confirming the adequacy of the item selection process. Therefore, the adjusted version of the scale is characterized by a more homogeneous structure, supported by both the Cronbach’s alpha values and the item–total statistics. The item–total statistics and Cronbach’s alpha values are presented in Table 18.

Analysis of variance (ANOVA) was used to examine the differences between the items included in the adjusted version of the scale. The results indicate the presence of statistically significant differences between items, with an F value of 7.414 (*p* < 0.001).

The variance associated with differences between items (Mean Square = 2.301) was higher than the residual variance (Mean Square = 0.310), suggesting that the statements included in the final version of the scale capture distinct aspects of the analyzed construct and are not evaluated uniformly by respondents. This differentiation is consistent with a coherent scale structure, in which items contribute complementarily to the measurement of the same dimension.

The overall mean score (Grand Mean = 4.01) indicates a generally high level of agreement with the included statements, which is consistent with the descriptive analysis results and with the improved Cronbach’s alpha values obtained after the scale adjustment.

A one-way ANOVA was performed to examine differences between the items included in the adjusted version of the scale, and the results are presented in Table 19.

The reliability of the adjusted version of the scale was further evaluated using the Intraclass Correlation Coefficient (ICC), calculated based on a two-way mixed-effects model, in which the effects associated with respondents were considered random, while the effects associated with items were considered fixed. A consistency definition was applied, which excludes the variance between measurements from the total variance calculation.

For the single measures, the ICC value was 0.530, with a 95% confidence interval ranging between 0.397 and 0.661. This result indicates a moderate level of consistency between items when they are analyzed individually, reflecting an acceptable variability of responses within the adjusted version of the scale.

For the average measures, the ICC value was 0.819, with a 95% confidence interval between 0.724 and 0.886, suggesting a good level of reliability for the composite scale score. This value is consistent with the Cronbach’s alpha coefficient obtained after scale adjustment and supports the use of the average item score in subsequent statistical analyses.

The associated F test was statistically significant (F = 5.514; *p* < 0.001), confirming that the ICC values are significantly different from zero and indicating the presence of a real consistency between the items of the adjusted version of the scale. The reliability of the adjusted scale was further assessed using the intraclass correlation coefficient (ICC), and the results are presented in Table 20.

*Section 6: Preferred Types of Prosthetic Restorations in Cases with Periodontally Compromised Abutment Teeth*

The internal consistency of Section 6 of the questionnaire was evaluated using Cronbach’s alpha coefficient. The obtained value (α = 0.795) indicates a good level of internal reliability, suggesting an adequate coherence of the items included in this dimension.

This section consists of six items that explore dentists’ preferences regarding the types of prosthetic restorations used in clinical situations involving periodontally compromised abutment teeth. The obtained Cronbach’s alpha value indicates that the items are sufficiently correlated with each other to measure a common construct, without indicating excessive redundancy or major inconsistencies among the items. The internal consistency of Section 6 was assessed using Cronbach’s alpha, and the results are presented in Table 21.

The descriptive statistical analysis of the items included in Section 6 shows mean values within a moderate range, between 3.35 and 3.80, suggesting a balanced positioning of respondents regarding prosthetic therapeutic preferences in cases involving periodontally compromised abutment teeth.

The highest mean value was recorded for the item referring to the avoidance of prosthetic restorations involving teeth with more than 50% periodontal support loss (M = 3.80), indicating a clear tendency toward caution in abutment tooth selection. Additionally, the items referring to the use of mixed restorations and the avoidance of including teeth with reduced periodontal support in extensive fixed prostheses showed similar mean values (M ≈ 3.50), suggesting a moderate preference for therapeutic solutions adapted to biological risk.

In contrast, the preference for implant-supported restorations combined with natural teeth recorded the lowest mean value (M = 3.35), reflecting greater variability in opinions regarding this treatment approach. The use of extended provisional restorations and the preference for conventional fixed prosthetic restorations showed similar mean values (M = 3.44), indicating moderate acceptance of these strategies in the context of periodontal risk assessment.

The standard deviations, ranging between 0.67 and 0.95, indicate a moderate dispersion of responses without pronounced extremes. This variability is consistent with the diversity of clinical situations and the professional experience of the respondents. Descriptive statistics for the items included in Section 6 are summarized in Table 22.

The item–total statistics analysis indicates an adequate contribution of most items to the internal consistency of Section 6. The corrected item–total correlation values range between 0.430 and 0.844, indicating a moderate to strong association between individual items and the total scale score.

The highest item–total correlation was observed for the item referring to the preference for combined prosthetic restorations (fixed–removable) in cases with reduced periodontal support (r = 0.844), suggesting a strong alignment of this item with the overall construct measured by the section. Additionally, the item regarding the use of extended provisional restorations showed a good correlation with the total score (r = 0.638), indicating the relevance of this aspect in prosthetic decision-making.

The items referring to the preference for conventional fixed restorations, the avoidance of including teeth with reduced periodontal support in extensive fixed prostheses, and the use of implant-supported restorations in combination with natural teeth recorded moderate item–total correlations (r ≈ 0.43–0.51), suggesting a consistent but less dominant contribution to the overall scale homogeneity.

The Cronbach’s alpha values if each item was deleted were, in all cases, lower than or very close to the alpha value obtained for the entire section (α = 0.795), ranging between 0.704 and 0.789. This result indicates that removing any of the items would not lead to a substantial improvement in the internal consistency of the scale, supporting the retention of all items in the final structure of Section 6 (Table 23).

Analysis of variance (ANOVA) was used to examine the differences between the items included in Section 6 of the questionnaire. The results indicate the presence of statistically significant differences between items, with an F value of 3.293 (*p* = 0.007).

The comparison of mean square values shows that the variance associated with differences between items (Mean Square = 1.250) is higher than the residual variance (Mean Square = 0.380), suggesting that the statements included in this section are perceived differently by respondents and capture distinct aspects of prosthetic preferences in the context of periodontal compromise.

The overall mean score (Grand Mean = 3.51) indicates a moderate level of agreement regarding the therapeutic options investigated, a result that is consistent with the individual item mean values and with the response dispersion observed in the descriptive analysis. The differences between the items included in Section 6 were analyzed using ANOVA, and the results are presented in Table 24.

The reliability of Section 6 of the questionnaire was further evaluated using the Intraclass Correlation Coefficient (ICC), calculated based on a two-way mixed-effects model, in which the effects associated with respondents were considered random, while the effects associated with items were considered fixed. A consistency definition was applied, which excludes the variance between measurements from the total variance calculation.

For the single measures, the obtained ICC value was 0.393, with a 95% confidence interval ranging between 0.277 and 0.526. This result indicates a low to moderate level of consistency between items when analyzed individually, reflecting a higher variability of responses, which can be explained by the diversity of therapeutic options and clinical situations addressed in this section.

For the average measures, the ICC value was 0.795, with a 95% confidence interval between 0.697 and 0.869, indicating a good level of reliability for the composite scale score. This value is consistent with the Cronbach’s alpha coefficient obtained for Section 6 and supports the use of the average item score in subsequent statistical and interpretative analyses.

The associated F test was statistically significant (F = 4.878; *p* < 0.001), confirming that the ICC values are significantly different from zero and indicating the presence of real consistency between the items of this section beyond random variation.

Overall, the intraclass correlation coefficient results support the reliability of Section 6 at the aggregated score level and confirm its suitability for evaluating preferences regarding the types of prosthetic restorations used in cases involving periodontally compromised abutment teeth, as seen in Table 25.

*Section 7: Implant-Supported Prosthetic Restorations—Attitudes and Clinical Practices*

The reliability of Section 7 was evaluated using Cronbach’s alpha coefficient. The obtained value (α = 0.812) reflects a satisfactory level of internal consistency, indicating a coherent relationship among the items that compose this dimension. This section includes seven items that investigate dentists’ attitudes and clinical practices regarding the use of implant-supported prosthetic restorations. The obtained alpha value indicates that the items are sufficiently correlated to measure a common construct, without indicating excessive redundancy or major inconsistencies.

Some items in Section 7 address closely related aspects of implant-supported therapy, such as treatment preference and perceived predictability. While these constructs are related, they reflect different dimensions of clinical evaluation. However, the presence of closely related items may contribute to increased internal consistency and should be considered when interpreting the results.

The obtained result supports the stability and homogeneity of this section, indicating that the instrument is appropriate for evaluating clinical behavior and perceptions associated with implant-supported prosthetic restorations. Therefore, Section 7 can be considered reliable and suitable for use in further statistical analyses and in the overall interpretation of the questionnaire results, as can be seen in Table 26.

The descriptive analysis of the items composing Section 7 reveals a response profile centered around moderate mean values, ranging between 3.15 and 3.74. This distribution suggests the presence of nuanced attitudes among respondents regarding the use of implant-supported prosthetic restorations, without indicating extreme positions.

Items referring to the functional advantages and long-term predictability of implant-supported restorations recorded the highest mean scores. In particular, the perception of superior predictability compared to restorations supported by natural teeth with compromised periodontal support was relatively frequently expressed by respondents (M = 3.74). Additionally, the acceptability of combining natural teeth and implants as prosthetic abutments, in carefully selected clinical situations, was evaluated favorably (M = 3.65).

In contrast, the statement referring to the systematic preference for fixed implant-supported restorations, regardless of the viability of teeth with reduced periodontal support, obtained the lowest mean value (M = 3.15), indicating a cautious approach and a tendency toward individualized treatment planning.

Implant-retained removable restorations, the exclusive use of implants to avoid periodontal risks, and the impact of previous experience with implant complications were evaluated with scores close to the overall mean (M ≈ 3.48–3.57). This suggests moderate variability in opinions, likely influenced by the clinical context, professional experience, and patient profile. Descriptive statistics for the items included in Section 7 are presented in Table 27.

The item–total statistics analysis indicates an overall adequate contribution of the items to the internal consistency of Section 7. The corrected item–total correlations ranged between 0.205 and 0.796, highlighting varying levels of alignment of the statements with the construct evaluated by this section.

The items reflecting the preference for implant-supported prosthetic restorations and the perception of superior long-term predictability showed the highest item–total correlations, indicating strong integration within the scale structure. In contrast, the item referring to the preference for restorations exclusively supported by implants recorded a lower correlation with the total score. However, its removal was not considered necessary, as its impact on Cronbach’s alpha did not justify the loss of conceptual relevance.

The Cronbach’s alpha values if items were deleted were, in most cases, close to or lower than the overall alpha value of the scale (α = 0.812), supporting the retention of all items in the final version of the section. Table 28 presents the item–total statistics and Cronbach’s alpha values if item deleted for Section 7.

The Intraclass Correlation Coefficient (ICC) revealed statistically significant differences between the items of Section 7 (F = 3.607; *p* = 0.002). The variance associated with differences between items was higher than the residual variance, indicating that the statements included captured distinct aspects of attitudes and clinical practices regarding implant-supported prosthetic restorations, as seen in Table 29.

The overall mean score (Grand Mean = 3.53) suggests a moderate level of agreement among respondents regarding the statements analyzed, consistent with the results of the descriptive analysis. The differences identified between items support the section’s ability to discriminate between various therapeutic options without affecting the internal coherence of the scale.

The reliability of Section 7 was evaluated using the Intraclass Correlation Coefficient (ICC), based on a two-way mixed-effects model with a consistency definition. For single measures, the ICC value was 0.381 (95% CI: 0.272–0.511), indicating a low to moderate level of consistency between items when analyzed individually.

For the average measures, the ICC value reached 0.812 (95% CI: 0.724–0.880), reflecting a good level of reliability for the composite scale score. The associated F test was statistically significant (F = 5.316; *p* < 0.001), confirming the presence of real consistency among the items included in this section.

Overall, the ICC results support the use of the average item score of Section 7 as a reliable indicator of attitudes and clinical practices regarding implant-supported prosthetic restorations (Table 30).

The suitability of the data for exploratory factor analysis was assessed using the Kaiser–Meyer–Olkin (KMO) measure and Bartlett’s test of sphericity. The obtained KMO value (0.709) indicates a satisfactory level of sampling adequacy, suggesting the presence of sufficient correlations between items to justify the application of factor analysis.

Bartlett’s test of sphericity was statistically significant (χ^2^ = 1413.717; df = 190; *p* < 0.001), confirming that the correlation matrix differs significantly from an identity matrix. This result supports the existence of an underlying latent structure in the analyzed data and justifies the use of exploratory factor analysis to assess the construct validity of the questionnaire.

Exploratory factor analysis (EFA) was performed using Principal Component Analysis (PCA) with Varimax rotation to examine the latent structure of the questionnaire. The analysis resulted in the extraction of eight components with eigenvalues greater than 1, which together explained a substantial proportion of the total variance. The rotated factor loading matrix indicated that most items loaded strongly (≥0.40) on a single factor, suggesting a generally well-defined factorial structure. Distinct clusters of items were identified, corresponding to relevant conceptual domains, including clinical decision-making factors, implant-oriented attitudes, preference for extraction and implant therapy, professional competence, conservative therapeutic approaches, prosthetic strategies, preference for implant-only restorations, and risk-avoidance behaviors.

Specifically, items related to interdisciplinary collaboration, patient expectations, and financial considerations loaded significantly on Factor 1, reflecting the multifactorial nature of clinical decision-making. Factor 2 grouped items related to positive attitudes toward implant-supported restorations, while Factor 3 reflected a clear preference for tooth extraction and implant placement. Factor 4 was defined by items reflecting perceived professional competence and interest in further training.

Factors 5–8 represented more specific dimensions, including conservative approaches (Factor 5), prosthetic strategy preferences (Factor 6), preference for implant-only restorations (Factor 7), and avoidance of using compromised teeth in prosthetic planning (Factor 8).

A single item demonstrated cross-loading (≥0.40 on two factors), suggesting partial conceptual overlap between implant-oriented attitudes and preference for implant-only restorations. However, this did not significantly affect the overall interpretability of the factor structure. The rotated factor loading matrix is presented in Table 31, with loadings ≥0.40 highlighted.

The communality analysis indicates high values of explained variance for all included items, with extraction values ranging between 0.734 and 0.914. These results show that a substantial proportion of the variance of each item is explained by the identified factorial structure.

Most items presented communalities above 0.80, suggesting very good representation within the factorial model. The highest values were observed for items related to the integration of teeth with periodontal mobility into prosthetic restorations, the preference for implant-supported fixed restorations, and the perceived long-term predictability of implant-supported restorations, indicating a strong alignment of these items with the extracted factors.

Items with relatively lower communality values, such as those related to patient expectations and the influence of financial costs, remained above the acceptable threshold, confirming their relevant contribution to the overall factorial structure.

Overall, the high level of communalities supports the adequacy of the item selection and confirms that the factorial structure consistently explains the variability of the investigated constructs. These results contribute to supporting the construction of the questionnaire. The results are illustrated in Table 32 below:

The analysis of total variance explained indicates the extraction of eight components with eigenvalues greater than 1, according to the Kaiser criterion. Together, these components explain 84.56% of the total variance of the data, suggesting a high capacity of the factorial model to capture the latent structure of the questionnaire.

The first component explained 25.38% of the total variance, followed by the second component (14.49%) and the third component (10.72%). The contributions of the remaining components were progressively lower, each explaining between 5.20% and 9.10% of the variance. This distribution indicates a multifactorial structure, in which several dimensions contribute meaningfully to explaining the variability of responses.

After rotation, the explained variance was redistributed more evenly among the components, without modifying the cumulative percentage of explained variance. This redistribution supports the interpretability of the extracted factors and indicates a more clearly defined factorial structure.

Overall, the high level of total explained variance confirms the adequacy of the factorial model and supports the construct validity of the questionnaire.

The interpretation of the unrotated component matrix must be performed with caution, as it represents an intermediate stage of the exploratory factor analysis and reflects only the raw correlations between items and components.

At this stage, most items present factor loadings distributed across multiple components, with cross-loadings being frequent, indicating a complex factorial structure that is not yet conceptually well defined. Items referring to the preference for implants, the preservation of periodontally compromised teeth, and decision-making factors associated with clinical and economic contexts are distributed across several components without forming clearly distinct groupings. This type of distribution is characteristic of the pre-rotation stage and does not allow for conceptual interpretations or the naming of factors.

Accordingly, based on the unrotated matrix, neither item elimination nor final evaluation of construct validity is justified at this stage. However, the obtained results support the existence of multiple latent dimensions and justify the application of factor rotation, which is necessary to clarify the structure, reduce cross-loadings, and facilitate the conceptual interpretation of the extracted factors, as illustrated in Table 33.

The application of Varimax rotation within the exploratory factor analysis led to the identification of a clear factorial structure composed of eight distinct factors, each reflecting relevant conceptual dimensions of the investigated prosthetic attitudes and clinical practices. The items grouped coherently into factors capturing, on the one hand, the influence of the decision-making context and interdisciplinary collaboration, as well as the role of patient expectations and financial constraints, and on the other hand, the therapeutic orientation toward implant-supported restorations and the perceived predictability of these treatments.

The factorial structure also highlights a dimension associated with favorable attitudes toward extraction and implant placement, distinct from the conservative orientation focused on maintaining periodontally compromised teeth under controlled conditions.

Other identified dimensions relate to perceived professional competence and interest in continuing education, cautious prosthetic strategies based on the use of provisional restorations, as well as strict abutment tooth selection criteria and the preference for implant-only restorations. The high factor loadings and the logical distribution of items, in the absence of significant cross-loadings, support the internal coherence of the model and confirm the construct validity of the questionnaire, demonstrating its ability to adequately capture the complexity of prosthetic decision-making in the context of periodontal compromise.

The component transformation matrix indicates that, through Varimax rotation with Kaiser normalization, the initial factors were reoriented to better correspond to the conceptual dimensions investigated by the questionnaire. This reorientation led to an optimal redistribution of variance, reducing overlap between dimensions and facilitating the clear delineation of factors associated with therapeutic orientation, professional competence, and decision-making factors.

The orthogonal nature of the rotation confirms the independence of the extracted factors, suggesting that each dimension measures a distinct aspect of the prosthetic decision-making process. In this context, the transformation matrix supports the stability of the factorial structure and confirms that the questionnaire is constructed on coherent and well-differentiated dimensions, thereby strengthening the construct validity of the instrument, illustrated in Table 34.

## 4. Discussion

The decision between preserving a periodontally compromised tooth and replacing it with a dental implant remains one of the most debated topics in contemporary prosthodontics and periodontology. Although implant therapy has become increasingly predictable and widely used, the preservation of natural teeth continues to represent a fundamental principle of dental practice. The present study is based on a relatively small sample size of 111 dentists, predominantly composed of early-career dentists, which limits the representativeness of the findings. Therefore, the results should be interpreted with caution and cannot be generalized to the entire population of Romanian dentists.

To the best of our knowledge, there are no studies specifically addressing the development and validation of a structured questionnaire designed to assess early-career dentists’ perspectives and clinical attitudes toward tooth preservation and implant therapy. Although previous publications have explored treatment preferences using survey-based or case-based approaches, these studies differ in design and do not involve validated instruments, which limits direct comparability with the present findings.

The results of the present study suggest that clinicians tend to adopt a balanced therapeutic philosophy, in which tooth preservation is preferred when the periodontal condition can be stabilized, while implant therapy is considered a reliable alternative in cases with unfavorable prognosis. The predominance of early-career dentists represents a design limitation of the current study, as the results do not fully reflect the clinical attitudes of more experienced Romanian clinicians.

This balanced approach is consistent with the classical periodontal–prosthetic concepts described in the literature, which emphasize that teeth with reduced periodontal support can function successfully for many years when periodontal inflammation is controlled and appropriate prosthetic stabilization is provided [18,19]. Long-term periodontal studies have demonstrated that tooth survival in treated periodontal patients can be comparable to the survival of implants in certain clinical conditions, particularly when patients are compliant with supportive periodontal therapy [20,21]. These findings support the idea that the extraction of compromised teeth should not be considered the default treatment option, but rather a decision based on individual prognosis and risk assessment.

The item addressing financial cost was designed to capture the perceived influence of economic factors on treatment decision-making, rather than to differentiate between specific cost components such as initial treatment cost and long-term maintenance cost. Therefore, the results reflect clinicians’ overall perception of financial impact, not an objective economic evaluation.

Some items included in Section 7 address closely related aspects of implant-supported therapy, which may result in a certain degree of overlap in responses. Although this does not indicate critical redundancy, it may contribute to increased covariance between items and should be considered when interpreting the internal consistency of the scale. Item–total correlation analysis did not indicate problematic redundancy, and the internal consistency coefficient remained within acceptable limits for exploratory research. Given the pilot nature of the study, all items were retained in order to preserve the conceptual coverage of the construction.

Future studies may further evaluate potential item redundancy and consider refinement of the scale where appropriate

At the same time, the increasing acceptance of implant therapy among clinicians can be explained by the high survival rates reported for implant-supported prosthetic restorations [22]. Systematic reviews and long-term clinical studies have consistently reported implant survival rates exceeding 90% over periods of 10 years or more, which has contributed to the perception of implant therapy as a predictable and efficient treatment option [23,24]. However, several authors have emphasized that implant survival should not be confused with implant success, as biological complications such as peri-implantitis remain a significant clinical concern [24,25]. Therefore, the decision to replace a tooth with an implant should consider not only survival rates but also biological and technical complications, maintenance requirements, and long-term patient-related factors [26].

Another important aspect highlighted by the present study is the complex and multifactorial nature of prosthetic treatment planning in cases involving periodontally compromised teeth. Clinical decision-making does not depend solely on the periodontal status of the tooth but also on factors such as patient age, financial considerations, patient expectations, clinician experience, and the possibility of interdisciplinary treatment [27]. Previous studies on clinical decision-making in dentistry have shown that treatment planning is influenced by a combination of biological, technical, economic, and behavioral factors, and that different clinicians may recommend different treatments for the same clinical situation [28,29]. This variability reflects the complexity of treatment planning rather than inconsistency in clinical reasoning.

Literature consistently emphasizes that interdisciplinary treatment planning improves clinical outcomes and allows more conservative therapeutic approaches in many complex cases [30]. Therefore, the decision between tooth preservation and implant placement should ideally be made within an interdisciplinary framework rather than from a single-specialty perspective.

The variability observed in prosthetic treatment preferences also reflects the absence of universally applicable clinical guidelines for managing teeth with reduced periodontal support used as prosthetic abutments. While clinical guidelines exist for periodontal treatment and implant therapy, fewer guidelines address the specific decision-making process between maintaining compromised teeth and replacing them with implants [31]. This may explain why clinicians rely heavily on clinical experience, personal training, and interdisciplinary consultation when making therapeutic decisions in such cases.

The questionnaire was designed to assess dentists’ self-reported perceptions, attitudes, and therapeutic preferences, and was not intended as a clinical decision-making tool based on predefined technical criteria. Therefore, it does not include specific clinical thresholds such as degree of furcation involvement, tooth mobility, or extent of bone loss.

While these parameters are essential in evidence-based clinical practice, their absence from the instrument limits its ability to directly evaluate decision-making against objective clinical standards. This represents a limitation of the study.

The factorial structure identified in the present study suggests that dentists’ decision-making regarding periodontally compromised teeth and implant therapy is not a single-dimensional construct but rather a multidimensional process involving conservative treatment philosophy, implant-oriented treatment philosophy, interdisciplinary decision-making, and prosthetic treatment strategy selection.

The present study has several limitations that should be considered when interpreting the results. The relatively small sample size of 111 and the predominance of participants with limited professional experience may influence the generalizability of the findings. In addition, the study evaluated self-reported attitudes rather than actual clinical behavior, and treatment decisions in real clinical situations may differ from theoretical preferences expressed in questionnaires.

The predominance of early-career dentists and the urban-based distribution of participants may introduce sampling bias and limit the generalizability of the findings.

The questionnaire was distributed as an open online survey based on voluntary participation, without controlled recruitment of participants. Consequently, a response rate could not be calculated, and the possibility of self-selection bias cannot be excluded.

In the present study, item refinement was performed using a combination of statistical indicators and conceptual considerations. As this process was partially data-driven and not fully pre-specified, it may introduce a risk of overfitting and should be interpreted with caution.

The intraclass correlation coefficient (ICC) was calculated using a two-way mixed-effects model with a consistency definition, in which respondents were treated as random effects and questionnaire items as fixed effects. This approach was selected because the instrument consists of a predefined and conceptually grounded set of items, developed to reflect specific clinical constructs relevant to the study objectives.

Given the exploratory and pilot nature of the study, this modeling approach is appropriate for preliminary validation. Future research may incorporate alternative analytical strategies, including models that treat items as random effects, particularly in the context of large-scale validation studies aimed at enhancing generalizability.

Another limitation of the study is the lack of a clearly defined subgroup of oral surgery specialists, whose clinical perspective may influence decision-making regarding tooth extraction and implant therapy. This reduced number of oral and maxillo-facial surgeons’ participation in the current study may be influenced by the current overlapping specialty particularities which exist in Romania regarding implant therapy.

The retention of eight factors based on the eigenvalue criterion may be associated with a risk of overextraction, which is a known limitation of this method, particularly in studies with relatively small sample sizes. Therefore, the identified factor structure should be interpreted with caution and considered exploratory.

Further analyses comparing responses according to dental specialty and years of professional experience were not performed in this pilot study due to the limited sample size and uneven distribution of participants. However, such subgroup analyses would provide valuable insights into the influence of professional background and clinical experience on therapeutic attitude and should be considered in future studies involving larger sample populations.

The use of an open online distribution strategy, without a defined sampling frame, prevented the calculation of a response rate and may have introduced selection bias, as participation was voluntary and self-selected.

The proportion of participants reporting academic involvement (18.9%) may therefore be higher than expected in the general population of practicing dentists, which could influence the overall response pattern toward a more guideline-oriented perspective. Accordingly, the findings of the present study should be interpreted as preliminary and should not be considered representative of the broader population of Romanian dentists. Future research is required to validate the instrument in larger and more representative samples, using controlled sampling approaches.

In addition, no bootstrapped or resampling-based stability analyses were performed for the factor structure, which may further limit the robustness of the exploratory factor analysis. This aspect should be addressed in future studies involving larger samples.

More robust methods for factor retention, such as parallel analysis, were not applied in the present study and should be considered in future research.

Additional analyses to assess convergent or divergent validity, including comparisons across demographic variables such as specialty or years of experience, were not performed due to the limited sample size. These analyses could provide further evidence of construct validity and should be considered in future studies.

The extraction of eight components explaining a high proportion of variance should be interpreted with caution, as the sample size in relation to the number of items may increase the risk of over-factoring.

A further limitation concerns the optimization of Section 5, where item removal was performed to improve internal consistency. Although this approach increased reliability, it may have reduced the breadth of the construction by excluding items related to clinical guidelines, which should be considered when interpreting the results.

Nevertheless, as a pilot study focused on questionnaire development and preliminary validation, the results provide useful information regarding the structure and psychometric properties of the instrument.

It should be noted that the present findings are based on self-reported attitudes and therapeutic preferences, which may not fully reflect actual clinical behavior. Therefore, further validation of the questionnaire is required, including behavioral or observational studies, as well as the assessment of test–retest reliability to evaluate the stability of responses over time.

Future development of the questionnaire may include the addition of clinical scenarios or parameter-based items in order to complement the current perception-based approach and to enable the assessment of guideline-oriented decision-making.

## 5. Conclusions

This pilot study resulted in the development and preliminary validation of a structured questionnaire with satisfactory psychometric properties, including good internal consistency and acceptable construct validity. This aspect confirms that the instrument can be used to investigate conservative orientation in prosthodontic clinical practice, but the interpretation of these results should take into account the predominance of early-career dentists with limited clinical experience within the study sample.

The findings indicate that dentists’ therapeutic preferences regarding tooth preservation versus implant therapy are influenced by multiple factors, including clinical considerations, professional experience, and individual perceptions.

The study also highlights the predominance of early-career Romanian dentists in the sample, which may influence the observed general trends but is in line with the study design. Although the results provide initial insights into dentists’ clinical attitudes, their generalizability is limited. This preliminary validated instrument offers a useful methodological tool for future studies involving larger and more diverse populations, in terms of experience and specialties.

## Figures and Tables

**Table 1:** Internal Consistency Analysis of Section 2—Attitudes Regarding the Use of Periodontally Compromised Teeth as Prosthetic Abutments.

Cronbach’s Alpha	N of Items
0.809	4

**Table 2:** Descriptive statistics for Section 2—Attitudes regarding the use of periodontally compromised teeth as prosthetic abutments.

Item	Mean	Std. Deviation
I consider that maintaining periodontally compromised teeth is a viable option in prosthetic rehabilitation when periodontal treatment is properly performed.	4.5185	0.57432
I prefer to preserve moderately periodontally compromised natural teeth rather than extracting them and replace them with dental implants.	4.4444	0.69137
The inclusion of teeth with Grade I–II periodontal mobility in prosthetic restorations is acceptable, provided they are stabilized through periodontal treatment.	4.5000	0.72032
I prefer a conservative therapeutic approach when the patient is compliant, maintains adequate oral hygiene, and agrees to regular follow-up.	4.7037	0.46091

**Table 3:** Item–total statistics for Section 2—Attitudes regarding the use of periodontally compromised teeth as prosthetic abutments.

Item	Scale Mean If Item Deleted	Scale Variance If Item Deleted	Corrected Item–Total Correlation	Cronbach’s Alpha If Item Deleted
I consider that maintaining periodontally compromised teeth is a viable option in prosthetic rehabilitation when periodontal treatment is properly performed.	13.6481	2.572	0.550	0.795
I prefer to preserve moderately periodontally compromised natural teeth rather than extract them and replace them with dental implants.	13.7222	2.129	0.648	0.752
The inclusion of teeth with Grade I–II periodontal mobility in prosthetic restorations is acceptable, provided they are stabilized through periodontal treatment.	13.6667	1.962	0.711	0.720
I prefer a conservative therapeutic approach when the patient is compliant, maintains adequate oral hygiene, and agrees to regular monitoring.	13.4630	2.706	0.657	0.765

**Table 4:** Analysis of Variance between Items (ANOVA) for Section 2 of the Questionnaire.

		Sum of Squares	df	Mean Square	F	*p*
Between People		51.875	53	0.979		
Within People	Between Items	2.051	3	0.684	3.660	0.014
	Residual	29.699	159	0.187		
	Total	31.750	162	0.196		
Total		83.625	215	0.389		

Grand Mean = 4.5417. df = degrees of freedom; *p* = statistical significance level.

**Table 5:** Intraclass correlation coefficients for Section 2 of the questionnaire.

	Intraclass Correlation	95% Confidence Interval		F Test with True Value 0			
		Lower Bound	Upper Bound	Value	df1	df2	*p*
Single Measures	0.515	0.380	0.648	5.240	53	159	<0.001
Average Measures	0.809	0.710	0.881	5.240	53	159	<0.001

df = degrees of freedom; *p* = statistical significance level.

**Table 6:** Internal Consistency Analysis of Section 3—Attitudes Regarding Tooth Extraction and Dental Implants.

Cronbach’s Alpha	N of Items
0.853	5

**Table 7:** Descriptive Statistics of the Items Included in Section 3 of the Questionnaire.

Item	Mean	Std. Deviation
Dental implants are preferable in most cases where the periodontium is severely affected.	3.4630	1.07656
Replacing teeth with dental implants provides more predictable long-term stability than maintaining a periodontally compromised tooth.	3.2963	0.96406
I consider implant placement more efficient in terms of time and outcomes than complex periodontal treatments.	3.0000	1.08158
The risk of peri-implantitis is lower than the risk of subsequent loss of a tooth affected by periodontal disease.	2.9630	1.02723

**Table 8:** Item–Total Statistics for Section 3 of the Questionnaire.

Item	Scale Mean If Item Deleted	Scale Variance If Item Deleted	Corrected Item–Total Correlation	Cronbach’s Alpha If Item Deleted
Dental implants are preferable in most cases where the periodontium is severely affected.	11.5370	13.008	0.528	0.858
Replacing teeth with dental implants provides more predictable long-term stability than maintaining a periodontally compromised tooth.	11.7037	12.326	0.740	0.806
I consider implant placement more efficient in terms of time and clinical outcomes than complex periodontal treatments.	12.0000	12.151	0.656	0.825
In my clinical practice, I prefer extraction and implant placement even in cases where the tooth could theoretically be preserved.	12.7222	11.374	0.657	0.828
The risk of peri-implantitis is lower than the risk of subsequent loss of a tooth affected by periodontal disease.	12.0370	11.734	0.778	0.794

**Table 9:** Analysis of Variance Between the Items of Section 3 of the Questionnaire (ANOVA Test).

		Sum of Squares	df	Mean Square	F	*p*
Between People		193.600	53	3.653		
Within People	Between Items	44.556	4	11.139	20.743	<0.001
	Residual	113.844	212	0.537		
	Total	158.400	216	0.733		
Total		352.000	269	1.309		

Grand Mean = 3.0000. df = degrees of freedom; *p* = statistical significance level.

**Table 10:** Intraclass Correlation Coefficient (ICC) for Section 3 of the Questionnaire.

	Intraclass Correlation	95% Confidence Interval		F Test with True Value 0			
		Lower Bound	Upper Bound	Value	df1	df2	*p*
Single Measures	0.537	0.415	0.661	6.802	53	212	<0.001
Average Measures	0.853	0.780	0.907	6.802	53	212	<0.001

df = degrees of freedom; *p* = statistical significance level.

**Table 11:** Internal Consistency Analysis of Section 4—Decision-Making Factors in Prosthetic Treatment Planning.

Cronbach’s Alpha	N of Items
0.859	6

**Table 12:** Descriptive statistics of the items included in Section 4 of the questionnaire.

Item	Mean	Std. Deviation
The individual prognosis of the tooth (mobility, attachment level, bone support) is a determining factor in the therapeutic decision.	4.1296	0.61572
The patient’s age significantly influences the choice between extraction and preservation of the abutment tooth.	3.7963	0.97863
Financial costs for the patient influence the final decision in the treatment plan.	4.2593	0.78151
The patient’s expectations and preferences regarding tooth preservation are always taken into consideration.	3.8704	0.89118
Collaboration with a periodontist influences the decision to preserve a compromised tooth.	4.1852	0.70240
Interdisciplinary collaboration (e.g., endodontist, periodontist, prosthodontist) influences the decision regarding extraction.	4.1296	0.72804

**Table 13:** Item–Total Statistics for Section 4 of the Questionnaire.

Item	Scale Mean If Item Deleted	Scale Variance If Item Deleted	Corrected Item–Total Correlation	Cronbach’s Alpha If Item Deleted
The individual prognosis of the tooth (mobility, attachment level, bone support) is a determining factor in the therapeutic decision.	20.2407	10.488	0.599	0.846
The patient’s age significantly influences the choice between extraction and preservation of the abutment tooth.	20.5741	8.702	0.623	0.847
Financial costs for the patient influence the final decision in the treatment plan.	20.1111	9.535	0.645	0.836
The patient’s expectations and preferences regarding tooth preservation are always taken into consideration.	20.5000	9.160	0.612	0.845
Collaboration with a periodontist influences the decision to preserve a compromised tooth.	20.1852	9.399	0.781	0.815
Interdisciplinary collaboration (e.g., endodontist, periodontist, prosthodontist) influences the decision regarding extraction.	20.2407	9.507	0.717	0.825

**Table 14:** Analysis of Variance among Items in Section 4 of the Questionnaire (ANOVA Test).

		Sum of Squares	df	Mean Square	F	*p*
Between People		117.099	53	2.209		
Within People	Between Items	9.210	5	1.842	5.920	<0.001
	Residual	82.457	265	0.311		
	Total	91.667	270	0.340		
Total		208.765	323	0.646		

df = degrees of freedom; *p* = statistical significance level.

**Table 15:** Intraclass Correlation Coefficient (ICC) for Section 4 of the Questionnaire.

	Intraclass Correlation	95% Confidence Interval		F Test with True Value 0			
		Lower Bound	Upper Bound	Value	df1	df2	*p*
Single Measures	0.504	0.388	0.628	7.101	53	265	<0.001
Average Measures	0.859	0.792	0.910	7.101	53	265	<0.001

df = degrees of freedom; *p* = statistical significance level.

**Table 16:** Analysis of Internal Consistency of the Scale Before and After Adjustment for Section 5.

Initial Cronbach’s Alpha	N of Items	Cronbach’s Alpha After	N of Items
0.548	7	0.819	4

**Table 17:** Comparison of descriptive statistics for the items in the initial and adjusted scale versions—Section 5.

Item	Initial Mean	Initial Std. Deviation	Adjusted Mean	Adjusted Std. Deviation
I feel competent to correctly evaluate the viability of a periodontally compromised tooth as an abutment.	3.9074	0.73378	4.1296	0.70165
I am confident in my clinical abilities to develop a prosthetic treatment plan on periodontally affected teeth.	3.7963	0.78619	–	–
I have received sufficient training in the preservation of teeth with compromised periodontium.	3.2222	0.98415	3.7963	0.91897
I believe there are gaps in clinical guidelines regarding the decision between extraction and tooth preservation.	3.5741	0.81500	3.8889	0.83929
I use the European Federation of Periodontology (EFP) S3 Guideline in my daily practice.	3.2407	0.97003	–	–
I know and apply the 2018 EFP classification in periodontal diagnosis.	3.4630	1.05889	–	–
I am interested in additional courses or training in periodontology applied to prosthetic dentistry.	4.3704	0.68118	4.2407	0.77545

**Table 18:** Comparison of item–total statistics and the impact of scale adjustment on internal consistency for Section 5.

Item	Scale Mean If Item Deleted	Scale Variance If Item Deleted	Corrected Item–Total Correlation	Cronbach’s Alpha If Item Deleted (Initial)	Cronbach’s Alpha If Item Deleted (Adjusted)
I feel competent to correctly evaluate the viability of a periodontally compromised tooth as an abutment.	21.6667	7.774	0.418	0.463	0.708
I am confident in my clinical abilities to develop a prosthetic treatment plan on periodontally affected teeth.	21.7778	7.421	0.463	0.440	–
I have received sufficient training in the preservation of teeth with compromised periodontium.	22.3519	7.327	0.324	0.488	0.787
I believe there are gaps in clinical guidelines regarding the decision between extraction and tooth preservation.	22.0000	10.264	−0.173	0.656	0.798
I use the European Federation of Periodontology (EFP) S3 Guideline in my daily practice.	22.3333	6.943	0.418	0.444	–
I know and apply the 2018 EFP classification in periodontal diagnosis.	22.1111	6.780	0.385	0.458	–
I am interested in additional courses or training in periodontology applied to prosthetic dentistry.	21.2037	8.920	0.157	0.547	0.795

**Table 19:** ANOVA analysis of the items included in the adjusted scale—Section 5.

		Sum of Squares	df	Mean Square	F	*p*
Between People		90.708	53	1.711		
Within People	Between Items	6.903	3	2.301	7.414	<0.001
	Residual	49.347	159	0.310		
	Total	56.250	162	0.347		
Total		146.958	215	0.684		

**Table 20:** Intraclass Correlation Coefficient (ICC) for the adjusted version of the scale for Section 5.

	Intraclass Correlation	95% Confidence Interval		F Test with True Value 0			
		Lower Bound	Upper Bound	Value	df1	df2	*p*
Single Measures	0.530	0.397	0.661	5.514	53	159	<0.001
Average Measures	0.819	0.724	0.886	5.514	53	159	<0.001

**Table 21:** Internal Consistency Analysis of Section 6—Preferred Types of Prosthetic Restorations.

Cronbach’s Alpha	N of Items
0.795	6

**Table 22:** Descriptive Statistics for the Items Included in Section 6 of the Questionnaire.

Item	Mean	Std. Deviation
In cases with periodontally compromised teeth, I prefer fixed prosthetic restorations (e.g., dental bridges).	3.4444	0.81650
I prefer combined prosthetic restorations (fixed–removable) in cases with reduced periodontal support.	3.5185	0.66562
I frequently use extended provisional restorations to evaluate the behavior of periodontally compromised teeth.	3.4444	0.79305
I prefer implant-supported restorations in combination with natural teeth.	3.3519	0.95478
I avoid including teeth with reduced periodontal support in extensive fixed prosthetic restorations.	3.5000	0.69364
I prefer prosthetic restorations that do not involve teeth with periodontal support loss greater than 50%.	3.7963	0.78619

**Table 23:** Item–Total Statistics for Section 6 of the Questionnaire.

Item	Scale Mean If Item Deleted	Scale Variance If Item Deleted	Corrected Item–Total Correlation	Cronbach’s Alpha If Item Deleted
In cases with periodontally compromised teeth, I prefer fixed prosthetic restorations (e.g., dental bridges).	17.6111	8.355	0.442	0.789
I prefer combined prosthetic restorations (fixed–removable) in cases with reduced periodontal support.	17.5370	7.574	0.844	0.704
I frequently use extended provisional restorations to evaluate the behavior of periodontally compromised teeth.	17.6111	7.676	0.638	0.742
I prefer implant-supported restorations in combination with natural teeth.	17.7037	7.533	0.509	0.779
I avoid including teeth with reduced periodontal support in extensive fixed prosthetic restorations.	17.5556	8.855	0.430	0.789
I prefer prosthetic restorations that do not involve teeth with periodontal support loss greater than 50%.	17.2593	8.196	0.510	0.772

**Table 24:** Analysis of Variance (ANOVA) for the Items Included in Section 6 of the Questionnaire.

		Sum of Squares	df	Mean Square	F	*p*
Between People		98.139	53	1.852		
Within People	Between Items	6.250	5	1.250	3.293	0.007
	Residual	100.583	265	0.380		
	Total	106.833	270	0.396		
Total		204.972	323	0.635		

Grand Mean = 3.5093.

**Table 25:** Intraclass Correlation Coefficient (ICC) for Section 6 of the Questionnaire.

	Intraclass Correlation )	95% Confidence Interval		F Test with True Value 0			
		Lower Bound	Upper Bound	Value	df1	df2	*p*
Single Measures	0.393	0.277	0.526	4.878	53	265	<0.001
Average Measures	0.795	0.697	0.869	4.878	53	265	<0.001

**Table 26:** Internal Consistency of Section 7—Implant-Supported Prosthetic Restorations.

Cronbach’s Alpha	N of Items
0.812	7

**Table 27:** Descriptive statistics for Section 7—Implant-supported prosthetic restorations.

Item	Mean	Std. Deviation
I prefer implant-supported prosthetic restorations over restorations supported by natural teeth with reduced periodontal support.	3.6481	0.73092
Implant-supported prosthetic restorations offer superior long-term predictability compared to restorations supported by natural teeth with reduced periodontal support.	3.7407	0.73164
Implant-retained removable restorations are indicated in extensive cases, especially in elderly patients.	3.4815	0.92636
The combination of natural teeth and implants as prosthetic abutments is acceptable in selected cases, with careful monitoring.	3.6481	0.95478
I prefer restorations exclusively supported by implants (without including natural teeth) to avoid the risk of periodontal tissue compromise.	3.5741	0.92353
Previous experience with complications (e.g., peri-implantitis) influences my decisions regarding implant treatments.	3.5000	1.17762

**Table 28:** Item–total statistics for Section 7—Implant-supported prosthetic restorations.

Item	Scale Mean If Item Deleted	Scale Variance If Item Deleted	Corrected Item–Total Correlation	Cronbach’s Alpha If Item Deleted
I prefer implant-supported prosthetic restorations over restorations supported by natural teeth with reduced periodontal support.	21.0926	15.595	0.796	0.756
Implant-supported prosthetic restorations offer superior long-term predictability compared to restorations supported by natural teeth with reduced periodontal support.	21.0000	16.302	0.658	0.776
I frequently opt for fixed implant-supported restorations regardless of the viability of teeth with reduced periodontal support.	21.5926	13.265	0.774	0.741
Implant-retained removable restorations are indicated in extensive cases, especially in elderly patients.	21.2593	15.818	0.549	0.787
The combination of natural teeth and implants as prosthetic abutments is acceptable in selected cases, with careful monitoring.	21.0926	15.935	0.509	0.794
I prefer restorations exclusively supported by implants (without including natural teeth) to avoid the risk of periodontal tissue compromise.	21.1667	18.255	0.205	0.842
Previous experience with complications (e.g., peri-implantitis) influences my decisions regarding implant treatments.	21.2407	14.752	0.507	0.800

**Table 29:** Analysis of Variance (ANOVA) for the Items Included in Section 7 of the Questionnaire.

		Sum of Squares	df	Mean Square	F	Sig
Between People		156.910	53	2.961		
Within People	Between Items	12.053	6	2.009	3.607	0.002
	Residual	177.090	318	0.557		
	Total	189.143	324	0.584		
Total		346.053	377	0.918		

Grand Mean = 3.5344.

**Table 30:** Intraclass Correlation Coefficient (ICC) for Section 7 of the Questionnaire.

	Intraclass Correlation	95% Confidence Interval		F Test with True Value 0			
		Lower Bound	Upper Bound	Value	df1	df2	*p*
Single Measures	0.381	0.272	0.511	5.316	53	318	<0.001
Average Measures	0.812	0.724	0.880	5.316	53	318	<0.001

**Table 31:** Rotated factor loading matrix (Varimax rotation).

Item	F1	F2	F3	F4	F5	F6	F7	F8
Preference for maintaining moderately periodontally compromised natural teeth					0.849			
Inclusion of teeth with Grade I–II periodontal mobility					0.896			
Implants are preferable in severe cases			0.608					
Long-term stability of implants			0.692					
Preference for extraction and implant placement			0.885					
Risk of peri-implantitis vs. tooth loss			0.820					
Collaboration with a periodontist	0.815							
Interdisciplinary collaboration	0.879							
Competence in evaluating tooth viability				0.875				
Interest in further training				0.899				
Use of extended provisional restorations						0.829		
Avoidance of extensive restorations on compromised teeth								0.886
Preference for implants over natural teeth		0.673					0.447	
Predictability of implant-supported restorations		0.834						
Fixed implant-supported restorations		0.806						
Removable implant-supported restorations		0.891						
Preference for implant-only restorations							0.853	
Patient expectations	0.768							
Financial costs	0.829							
Preference for fixed restorations on natural teeth						0.792		

Factor loadings ≥ 0.40 are presented in bold. One item showed cross-loading (Factor 2 and Factor 7), indicating partial overlap between implant-oriented attitudes and preference for implant-exclusive restorations.

**Table 32:** Communalities (Exploratory Factor Analysis).

Item	Initial	Extraction
I prefer to maintain moderately periodontally compromised natural teeth rather than extract them and place implants.	1.000	0.840
The integration of teeth with Grade I–II periodontal mobility into prosthetic restorations is acceptable, provided they are stabilized through periodontal treatment.	1.000	0.906
Dental implants are preferable in most cases where periodontal support is severely compromised.	1.000	0.798
Replacement with dental implants provides more predictable long-term stability than maintaining a periodontally compromised tooth.	1.000	0.841
In my practice, I prefer extraction and implant placement even in cases where the tooth could theoretically be saved.	1.000	0.878
The risk of peri-implantitis is lower than the risk of future loss of a periodontally compromised tooth.	1.000	0.800
Collaboration with a periodontist influences the decision to preserve a compromised tooth.	1.000	0.858
Interdisciplinary collaboration (e.g., endodontist, periodontist, prosthodontist) influences the decision regarding extraction.	1.000	0.828
I feel competent to correctly evaluate the viability of a periodontally compromised tooth as an abutment.	1.000	0.870
I am interested in additional courses or training in periodontology applied to prosthetic dentistry.	1.000	0.892
I frequently use extended provisional restorations to evaluate the behavior of periodontally compromised teeth.	1.000	0.846
I avoid including teeth with reduced periodontal support in extensive fixed prosthetic restorations.	1.000	0.868
I prefer implant-supported prosthetic restorations over restorations supported by natural teeth with reduced periodontal support.	1.000	0.842
Implant-supported prosthetic restorations offer superior long-term predictability compared to restorations supported by natural teeth with reduced periodontal support.	1.000	0.881
I frequently opt for fixed implant-supported restorations regardless of the viability of teeth with reduced periodontal support.	1.000	0.914
Implant-retained removable restorations are indicated in extensive cases, especially in elderly patients.	1.000	0.848
I prefer restorations exclusively supported by implants (without including natural teeth) to avoid the risk of periodontal tissue compromise.	1.000	0.850
Patient expectations and preferences regarding tooth preservation are always taken into consideration.	1.000	0.734
Financial costs for the patient influence the final treatment plan decision.	1.000	0.767
In cases with periodontally compromised teeth, I prefer fixed prosthetic restorations (e.g., dental bridges).	1.000	0.853

Extraction Method: Principal Component Analysis.

**Table 33:** Unrotated Component Matrix from the Exploratory Factor Analysis.

Item	1	2	3	4	5	6	7	8
I prefer to maintain moderately periodontally compromised natural teeth rather than extract them and place implants.	−0.286	0.183	0.433	−0.262	0.666	0.128	0.080	0.045
The integration of teeth with Grade I–II periodontal mobility into prosthetic restorations is acceptable, provided they are stabilized through periodontal treatment.	−0.273	0.389	0.500	0.088	0.497	0.390	0.107	0.112
Dental implants are preferable in most cases where periodontal support is severely compromised.	0.558	0.229	0.138	−0.203	−0.111	0.598	0.022	0.054
Replacement with dental implants provides more predictable long-term stability than maintaining a periodontally compromised tooth.	0.747	0.383	0.101	−0.214	−0.035	0.251	0.102	−0.076
In my practice, I prefer extraction and implant placement even in cases where the tooth could theoretically be saved.	0.360	0.451	0.047	−0.396	−0.388	−0.161	0.388	0.245
The risk of peri-implantitis is lower than the risk of future loss of a periodontally compromised tooth.	0.617	0.350	0.066	−0.363	−0.300	−0.063	0.006	0.260
Collaboration with a periodontist influences the decision to preserve a compromised tooth.	0.697	−0.559	0.208	−0.034	−0.028	0.035	0.047	0.102
Interdisciplinary collaboration (e.g., endodontist, periodontist, prosthodontist) influences the decision regarding extraction.	0.513	−0.628	0.391	0.071	−0.041	0.036	−0.072	0.067
I feel competent to correctly evaluate the viability of a periodontally compromised tooth as an abutment.	0.243	0.494	0.301	0.487	−0.370	0.114	−0.124	−0.273
I am interested in additional courses or training in periodontology applied to prosthetic dentistry.	0.254	0.394	0.497	0.379	−0.141	−0.121	0.027	−0.495
I frequently use extended provisional restorations to evaluate the behavior of periodontally compromised teeth.	−0.315	0.097	0.501	0.458	−0.208	−0.156	−0.003	0.456
I avoid including teeth with reduced periodontal support in extensive fixed prosthetic restorations.	−0.137	−0.376	0.373	0.264	0.035	−0.156	0.687	0.031
I prefer implant-supported prosthetic restorations over restorations supported by natural teeth with reduced periodontal support.	0.727	0.136	0.085	0.257	0.218	−0.394	0.004	0.141
Implant-supported prosthetic restorations offer superior long-term predictability compared to restorations supported by natural teeth with reduced periodontal support.	0.759	0.009	−0.259	0.387	0.169	0.234	0.019	0.071
I frequently opt for fixed implant-supported restorations regardless of the viability of teeth with reduced periodontal support.	0.735	0.263	−0.308	0.094	0.410	−0.170	0.014	0.053
Implant-retained removable restorations are indicated in extensive cases, especially in elderly patients.	0.467	0.065	−0.453	0.562	0.213	0.046	−0.044	0.232
I prefer restorations exclusively supported by implants (without including natural teeth) to avoid the risk of periodontal tissue compromise.	0.221	0.437	0.257	−0.294	0.285	−0.510	−0.307	−0.151
Patient expectations and preferences regarding tooth preservation are always taken into consideration.	0.542	−0.400	0.400	−0.237	0.093	−0.182	−0.128	0.077
Financial costs for the patient influence the final treatment plan decision.	0.312	−0.590	0.337	−0.041	−0.072	0.159	−0.417	−0.047
In cases with periodontally compromised teeth, I prefer fixed prosthetic restorations (e.g., dental bridges).	−0.499	0.375	0.183	0.130	−0.130	0.035	−0.403	0.482

**Table 34:** Component Transformation Matrix.

Component	1	2	3	4	5	6	7	8
1	0.495	0.592	0.493	0.192	−0.141	−0.291	0.127	−0.062
2	−0.659	0.127	0.433	0.384	0.258	0.161	0.248	−0.247
3	0.485	−0.371	0.110	0.423	0.479	0.327	0.180	0.261
4	−0.073	0.527	−0.476	0.480	−0.114	0.356	−0.249	0.240
5	−0.014	0.388	−0.362	−0.295	0.686	−0.238	0.321	0.027
6	0.073	0.000	0.099	0.077	0.428	−0.142	−0.805	−0.358
7	−0.265	0.016	0.304	−0.064	0.106	−0.282	−0.253	0.823
8	0.070	0.258	0.312	−0.556	0.079	0.706	−0.116	0.056

## Data Availability

The datasets generated and analyzed during the current study are available from the corresponding author on reasonable request. The dataset is fully anonymized.

## Abbreviations

The following abbreviations are used in this manuscript:

ICC	Intraclass Correlation Coefficient
EFA	Exploratory Factor Analysis
PCA	Principal Component Analysis
KMO	Kaiser–Meyer–Olkin
